# A latent profile analysis of health behaviors among rural patients with ischemic stroke in China

**DOI:** 10.3389/fpubh.2026.1838937

**Published:** 2026-05-28

**Authors:** Yao Li, Xiaofan Fang, Siqi Ding, Zezhou Wang, Xue Dong, Aiping Wang

**Affiliations:** 1 The First Affiliated Hospital of China Medical University, Shenyang, Liaoning, China; 2 Xuzhou Medical University, Xuzhou, Jiangsu, China; 3 HanDan Central Hospital, Handan, Hebei, China; 4 Tangdu Hospital, Fourth Military Medical University, Xi'an, Shaanxi, China

**Keywords:** health behaviors, health knowledge, ischemic stroke, latent profile analysis, self-efficacy

## Abstract

**Background:**

Health behaviors are critical determinants of stroke recurrence risk and long-term clinical outcomes in patients with chronic diseases. For rural patients with ischemic stroke (IS) in China, understanding the heterogeneity of these behaviors is essential for developing targeted interventions to improve both behavioral adherence and subsequent quality of life.

**Methods:**

This cross-sectional study used convenience sampling to recruit 378 rural patients with ischemic stroke from five hospitals in Liaoning Province, China. Data were collected using the Socio-demographic Questionnaire, Stroke Health Knowledge Scale, Self-Efficacy for Managing Chronic Disease 6-Item Scale, and Health Behaviors Scale for Stroke Patients. Latent profile analysis (LPA) was conducted to identify behavioral subgroups, and multinomial logistic regression was used to examine correlates of profile membership.

**Results:**

Three distinct health behavior profiles were identified: “Low Health Behaviors Group” (34.13%), “Medium Health Behaviors Group” (50.00%), and “High Health Behaviors Group” (15.87%). Male gender was associated with higher odds of belonging to the Low and Medium Health Behaviors Groups (both *P* < 0.001); higher self-efficacy (OR = 0.29–0.34) and higher health knowledge (OR = 0.51–0.52) were associated with lower odds of belonging to unfavorable profiles (all *P* < 0.001).

**Conclusion:**

Health behaviors among rural Chinese IS survivors are heterogeneous. A majority (84%) exhibit suboptimal behavioral patterns, which are known correlates of higher stroke recurrence rates. The strong association of modifiable factors like self-efficacy and health knowledge with favorable profiles provides clear targets for secondary prevention interventions. Tailored strategies that address these factors are essential to mitigate stroke-related health disparities in this underserved population.

## 1 Introduction

Ischemic stroke (IS), an acute cerebrovascular event, primarily results from the narrowing or occlusion of cerebral arteries, leading to localized brain tissue necrosis due to interrupted blood supply (1). IS accounts for approximately 80% of all stroke cases and is characterized by high incidence, recurrence, mortality, and disability rates (2). According to the China Stroke Prevention and Control Report (2023), there are over 8 million existing IS patients in China, with approximately 1.8 million new cases annually, making IS the leading cause of death and disability among Chinese residents. Its 5-year recurrence rate reaches as high as 41% (3, 4).

Among various risk factors for IS, behavioral and lifestyle factors represent the only modifiable and controllable elements. Promoting healthy behaviors and modifying unhealthy lifestyles are recognized as the most cost-effective strategies for reducing stroke recurrence (5). Compelling evidence indicates that comprehensive behavioral management can substantially reduce stroke recurrence risk. Compelling evidence indicates that sustained adherence to health behaviors can reduce the risk of ischemic stroke recurrence by 40%−50% (6).

In rural China, patients with ischemic stroke face compounded challenges, including lower economic status, scarce healthcare resources, limited health awareness, inadequate family and community support, and persistently low self-efficacy (7–9). Epidemiological data reveal a striking urban-rural divide: rural residents experience a 1.8-fold higher 5-year recurrence rate and a 2.1-fold increased risk of disability after an ischemic stroke compared to urban residents (10, 11). Moreover, over 90% of rural patients return home for self-management after discharge, confronting a healthcare landscape characterized by professional shortages, geographical isolation, and financial constraints (12). These structural limitations lead to progressively declining adherence to health behaviors over time (13).

Effective health behavior management, encompassing consistent medication adherence, dietary modification, physical activity, and smoking cessation, serves as the cornerstone of secondary stroke prevention. More importantly, sustained engagement in these behaviors is inextricably linked to better physical function, psychological wellbeing, and overall health-related quality of life. However, real-world adherence among rural IS survivors remains alarmingly suboptimal. Epidemiological reports indicate that fewer than 40% of patients maintain prescribed medication regimens (14, 15), and less than 20% engage in regular physical exercise (16). This substantial gap between evidence-based recommendations and actual practice underscores both the urgent need and considerable potential for improving behavioral management in this vulnerable population (17).

Most previous research on health behaviors in stroke populations has adopted a variable-centered approach, focusing on average relationships across populations and examining factors in isolation. This paradigm fails to capture the heterogeneity within rural communities and overlooks individual differences in behavioral patterns, thereby hindering the development of precise intervention strategies. In contrast, person-centered approaches such as latent profile analysis (LPA) are designed to identify homogeneous subgroups (profiles) based on individuals' response patterns across multiple indicators (18). LPA has been applied to other stroke populations (e.g., examining risk factors or anxiety) (19, 20) and to health behaviors in at-risk groups (21). Yet, few studies have applied LPA to model the holistic profiles of multiple health behaviors specifically among rural Chinese ischemic stroke survivors.

Within this context, the roles of key facilitators like health knowledge and self-efficacy remain unclear at the profile level. Health knowledge provides the essential cognitive foundation (22), while self-efficacy reflects confidence in managing health behaviors (23). Particularly in resource-limited settings, higher self-efficacy might be crucial for overcoming barriers. However, their distinct contributions to predicting membership in specific health behavior profiles have not been fully elucidated. Clarifying such profile-specific influences is a crucial prerequisite for designing individualized interventions aimed at not only preventing recurrence but also preserving and enhancing patients' quality of life.

To address these gaps, this study had three aims: (1) to identify latent profiles of health behaviors among rural patients with ischemic stroke in China; (2) to compare sociodemographic characteristics across these profiles; and (3) to examine how health knowledge and self-efficacy are associated with profile membership. The findings are expected to inform health policy and targeted secondary prevention programs aimed at reducing the disproportionately high recurrence rates of ischemic stroke in rural China.

## 2 Methods

### 2.1 Participants and samples

#### 2.1.1 Study Design and Sample Size Consideration

This cross-sectional study employed convenience sampling. Although a formal *a priori* power calculation for latent profile analysis (LPA) is not standardized, the adequacy of our sample size was planned and justified based on established methodological recommendations. First, simulation studies suggest that a sample size of at least 300 is generally sufficient to obtain stable LPA solutions with accurate profile classification (24). Second, for the subsequent multinomial logistic regression, a common guideline recommends a minimum of 10 events per predictor variable (25). Our target sample size was set to exceed these thresholds to ensure robust estimates.

#### 2.1.2 Recruitment Procedure and Response

Eligible participants were recruited from the neurology outpatient clinics of five hospitals in Liaoning Province, China between January 20, 2025, and August 28, 2025. Clinical nurses assisted in identifying potential participants, after which a researcher explained the study and invited participation.

A total of 400 eligible patients were approached. Of these, 378 completed the survey, yielding a response rate of 94.5%. Twenty-two individuals declined or withdrew due to time constraints or personal reasons. All participants provided written informed consent. For those requiring assistance, researchers read the questionnaire items aloud and recorded responses based strictly on the patient's own answers. No questionnaires were excluded due to incompleteness.

#### 2.1.3 Inclusion and exclusion criteria

From the respondents, participants were included if they met all of the following criteria: age ≥18 years; diagnosis of ischemic stroke confirmed by cranial CT or MRI and meeting the criteria outlined in the Diagnostic Criteria for Various Cerebrovascular Diseases of the Chinese Medical Association (26) and the 2018 Chinese Guidelines for the Diagnosis and Treatment of Acute Ischemic Stroke (27); continuous residence in a rural area (defined as townships or villages according to China's administrative division system) for ≥1 year, with a concurrent agricultural household registration (Hukou); possession of basic language communication and comprehension abilities.

Participants were excluded if they met any of the following criteria: presence of other severe chronic illnesses (e.g., malignant tumors, heart failure, respiratory failure); non-rural permanent residents; or severe cognitive impairment, defined as a score of ≤ 17 on the Mini-Mental State Examination (MMSE) administered during the screening process. This cutoff has been validated for low-educated rural Chinese populations (28, 29).

### 2.2 Instruments

#### 2.2.1 Socio-demographic characteristics questionnaire

After a literature review and group discussion, the questionnaire was developed to collect the general socio-demographic characteristics of the participants. The questionnaire covers gender, age, occupation, marital status, and monthly income.

#### 2.2.2 Stroke health knowledge scale

The Stroke Health Knowledge Scale (SHKS) was developed by renowned Chinese scholar Wan Lihong et al. (30). It comprises 10 dimensions and 25 items, designed to accurately measure knowledge levels associated with stroke occurrence. The questionnaire covers 10 topics, including medication adherence and smoking cessation, stroke warning signs and exercise, stroke management, and nutrition. Typically, responses are categorized as “Yes,” “No,” or “Unsure.” Correct answers receive 1 point, while incorrect answers or responses of “Unsure” receive 0 points. Higher scores indicate more satisfactory health knowledge levels. The questionnaire's Cronbach's α coefficient is 0.87.

#### 2.2.3 Self-efficacy for managing chronic disease 6-item scale

The Self-Efficacy for Managing Chronic Disease Scale (SEMCD) was originally developed by the Patient Education Center at Stanford University in the United States. Lorig et al. (31) simplified the initial 36-item Chronic Disease Self-Efficacy Scale (CDSES) into a 6-item version, hence also known as the Self-Efficacy for Managing Chronic Disease 6-Item Scale (SEMCD6). Zhang Meixia (32) conducted cross-cultural adaptation and reliability/validity testing in China. This study employed the Chinese version of the Chronic Disease Management Self-Efficacy Scale (C-SEMCD6) for data collection. The scale comprises 6 items across 2 dimensions: the first 4 items assess symptom management self-efficacy, while the latter 2 items evaluate disease management self-efficacy. Each item is rated on a 1–10 scale, representing increasing confidence levels from “no confidence” to “complete confidence”. The total score is the average of the 6 items. The Cronbach's α coefficient for the scale ranges from 0.88 to 0.95.

#### 2.2.4 Health behaviors scale for stroke patients

The Health Behaviors Scale for Stroke Patients (HBS-SP) was formally developed by Wan Lihong et al. (33) in 2017. This scale comprises six dimensions: exercise, responsibility, adherence, nutrition, tobacco & alcohol and medication compliance. It consists of 25 items, each scored on a 5-point Likert scale ranging from 0 to 4 (0 = never, 1 = rarely, 2 = sometimes, 3 = often, 4 = always). The content validity index of this scale is 0.85, and the Cronbach's alpha coefficient for the total scale is 0.88. Higher total scores indicate more favorable health behaviors.

### 2.3 Data collection

With the assistance of clinical nurses, rural patients with ischemic stroke meeting inclusion criteria were recruited from outpatient clinics to participate in a questionnaire survey. After fully understanding the purpose and significance of the study, patients voluntarily and anonymously participated in this research. Ultimately, 94.50% (378 patients) participated in this study. For participants who had difficulty completing the questionnaire independently, trained researchers provided one-on-one assistance. To ensure consistency and minimize interviewer bias, a standardized protocol was followed: researchers read each question and all response options verbatim from the questionnaire without paraphrasing, adding explanations, or offering cues. Responses were recorded exactly as stated by the participant. All completed questionnaires underwent thorough on-site verification to ensure the accuracy and authenticity of the results. Any missing or incomplete responses were identified during verification, and participants were asked to complete the omitted items immediately. Consequently, no questionnaires had missing data, and no statistical imputation was required.

### 2.4 Data analysis

The analysis was carried out with Mplus 8.3 and SPSS 26.0 (IBM Corp). Categorical data were summarized using frequencies and percentages, whereas continuous measures following a normal distribution were described as mean ± standard deviation. Latent Profile Analysis (LPA) was implemented in Mplus 8.3 to uncover unobserved subgroups in the study population. Latent profile analysis (LPA) was conducted to categorize rural patients with ischemic stroke based on their scores across six health behavior dimensions (exercise, responsibility, adherence, nutrition, tobacco & alcohol and medication compliance), while self-efficacy and health knowledge were included as covariates in the multinomial regression analysis. The optimal model was selected guided by information criteria, comprising the Akaike Information Criterion (AIC), Bayesian Information Criterion (BIC), and the sample-size-adjusted BIC (aBIC), where smaller values are indicative of a superior model fit (34). The relative fit of the competing models was further evaluated using the Lo-Mendell-Rubin adjusted likelihood ratio test (LMR-LRT) and the bootstrap likelihood ratio test (BLRT). A statistically significant *p*-value (*p* < 0.05) for these tests justified the retention of the model with more latent profiles. Profile classification accuracy was assessed using entropy, which ranges from 0 to 1. Values of 0.80 or above suggest high confidence in assignments, typically corresponding to a classification accuracy of at least 90% (35).

We tested models with 2 to 5 latent profiles (5 as the maximum, as models with more classes typically become unstable and difficult to interpret). The following criteria were used to select the optimal model: (a) no class with less than 5% of the total sample to ensure stability; (b) significant LMR-LRT and BLRT (*p* < 0.05); (c) entropy ≥0.80; and (d) meaningful clinical interpretability of the profiles. We employed a standard three-step approach (36) for covariate inclusion: latent profiles were first estimated without covariates, then profile membership was regressed on self-efficacy and health knowledge using multinomial logistic regression while accounting for classification error. This approach avoids altering the profile solution while allowing examination of covariate effects. Multicollinearity was assessed using variance inflation factors (VIFs).

Following finalization of the latent profile model, the derived profile classifications were transferred to SPSS 26.0 for subsequent statistical procedures. The relationships among health knowledge, self-efficacy, and health behaviors were examined using Pearson's correlation coefficients. To investigate demographic variations across the identified profiles, continuous variables were evaluated by one-way ANOVA while categorical variables were assessed through chi-square tests. Furthermore, a multinomial logistic regression analysis was conducted to determine significant correlates of profile membership. Given the cross-sectional design, all regression results are interpreted as descriptive associations, not causal effects. For all statistical tests, significance was defined as a two-tailed *p*-value < 0.05.

## 3 Results

### 3.1 Participant characteristics

This study enrolled 378 rural patients diagnosed with ischemic stroke. As summarized in Table 1, the cohort consisted of 215 (56.88%) males and 163 (43.12%) females, with a mean age of 66.15 years (SD = 9.42). Among the participants, 237 (62.69%) were unemployed or physical labor, and the majority (73.81%) were married. The distribution of participants across sociodemographic characteristics for the comparative results across the three latent profiles are presented in Table 1.

**Table 1:** Participant characteristics (*N* = 378).

Variables	Category 1 (*n* = 129, 34.13%)	Category 2 (*n* = 189, 50%)	Category 3 (*n* = 60, 15.87%)	*χ^2^*	*P*
Gender	
Male	105 (81.4%)	99 (52.4%)	11 (18.3%)	69.52	< 0.001
Female	24 (18.6%)	90 (47.6%)	49 (81.7%)		
Occupation	
Unemployed	58 (45.0%)	43 (22.8%)	4 (6.7%)	37.31	< 0.001
Physical labor	36 (27.9%)	70 (37.0%)	26 (43.3%)		
Mental labor	18 (14.0%)	47 (24.9%)	21 (35.0%)		
Others	17 (13.2%)	29 (15.3%)	9 (15.0%)		
Marital status	
Single	0 (0.0%)	2 (1.1%)	0 (0.0%)	3.53	0.74
Married	110 (85.3%)	167 (88.4%)	51 (85.0%)		
Divorced	4 (3.1%)	5 (2.6%)	2 (3.3%)		
Widowed	15 (11.6%)	15 (7.9%)	7 (11.7%)		
Monthly income (RMB)	
< 1,000	64 (49.6%)	50 (26.5%)	35 (58.3%)	58.91	< 0.001
1,001~3,000	25 (19.4%)	96 (50.8%)	9 (15.0%)		
3,001~6,000	27 (20.9%)	35 (18.5%)	6 (10.0%)		
>6,000	13 (10.1%)	8 (4.2%)	10 (16.7%)		

Category 1: Low Health Behaviors Group; Category 2: Medium Health Behaviors Group; Category 3: High Health Behaviors Group.

The descriptive statistics (e.g., means, standard deviations) for the scores on the Health Knowledge, Self-Efficacy, and Health Behavior scales are summarized in Table 2.

**Table 2:** The score of key variables (*N* = 378).

Items	Total score (x ±s)	Average score (x ±s)
**Health behaviors**	52.11 ± 18.99	2.08 ± 0.76
Exercise	12.48 ± 5.10	2.08 ± 0.85
Responsibility	5.68 ± 2.70	1.89 ± 0.90
Adherence	8.46 ± 3.51	2.11 ± 0.88
Nutrition	12.97 ± 5.09	2.16 ± 0.85
Tobacco & alcohol	4.12 ± 1.83	2.06 ± 0.92
Medication compliance	8.40 ± 3.65	2.10 ± 0.91
**Health knowledge**	17.65 ± 6.20	0.71 ± 0.25
**Self-efficacy**	34.97 ± 9.06	5.83 ± 1.51

### 3.2 Correlation among variables

As presented in Table 3, bivariate correlation analysis revealed that all three key variables were significantly and positively intercorrelated (*P* < 0.01), with coefficient magnitudes ranging from moderate to strong.

**Table 3:** Correlation among variables (*N* = 378).

Variables	Health knowledge	Self-efficacy	Health behaviors
Health knowledge	1		
Self-efficacy	0.527^**^	1	
Health behaviors	0.452^**^	0.523^**^	1

^**^means *P* < 0.01.

### 3.3 Characteristics and naming of latent profiles for health behaviors

In the potential profile analysis, this study fitted a total of 2–5 models using the 6 dimensions of stroke health behaviors score as exogenous variables, and the fitting indexes of each model are shown in Table 4. Three-category models were selected as the best classification number, with low AIC, BIC, and aBIC values, indicating high model fitting excellence; the *p*-values of LMR and BLRT were both less than 0.01, showing their categorical significance; entropy values were high up to 0.910, reflecting the high accuracy of classification; The average latent class posterior probabilities for the three classes were 0.945 (Low Health Behaviors Group), 0.958 (Medium Health Behaviors Group), and 0.999 (High Health Behaviors Group), all well above the recommended threshold of 0.80, indicating excellent classification accuracy. The minimum number of category samples is 60, accounting for 15.87% of the total samples, ensuring the stability of the results. Although fit indices kept decreasing for models with more classes, the four- and five-class models were rejected because their LMR-LRT values became non-significant (*p* = 0.247 and *p* = 0.289, respectively), indicating no statistical improvement over the three-class model. Moreover, their smallest classes (6.35% and 6.88%) were deemed too small for stable, meaningful interpretation. In conclusion, the 3-category model is the best choice because of its excellent performance in terms of goodness-of-fit, classification significance, accuracy and stability.

**Table 4:** Indexes for fitting the model of LPA in health behaviors (*N* = 378).

Model	AIC	BIC	aBIC	LMR (*p*)	BLRT (*p*)	Entropy	Minimum model sample size (%)
2	4,466.921	4,541.684	4,481.402	0.000	0.000	0.980	68 (17.99%)
3	4,021.293	4,123.600	4,041.108	0.001	0.000	0.910	60 (15.87%)
4	3,942.078	4,071.930	3,967.228	0.247	0.000	0.893	24 (6.35%)
5	3,761.949	3,919.344	3,792.433	0.289	0.000	0.965	26 (6.88%)

aBIC, sample-size Adjusted Bayesian Information Criterion; AIC, Akaike Information Criterion; BIC, Bayesian Information Criterion; BLRT, Bootstrapped Likelihood Ratio Test; LMR, Lo–Mendell–Rubin Adjusted Likelihood Ratio Test.

**Figure 1:**
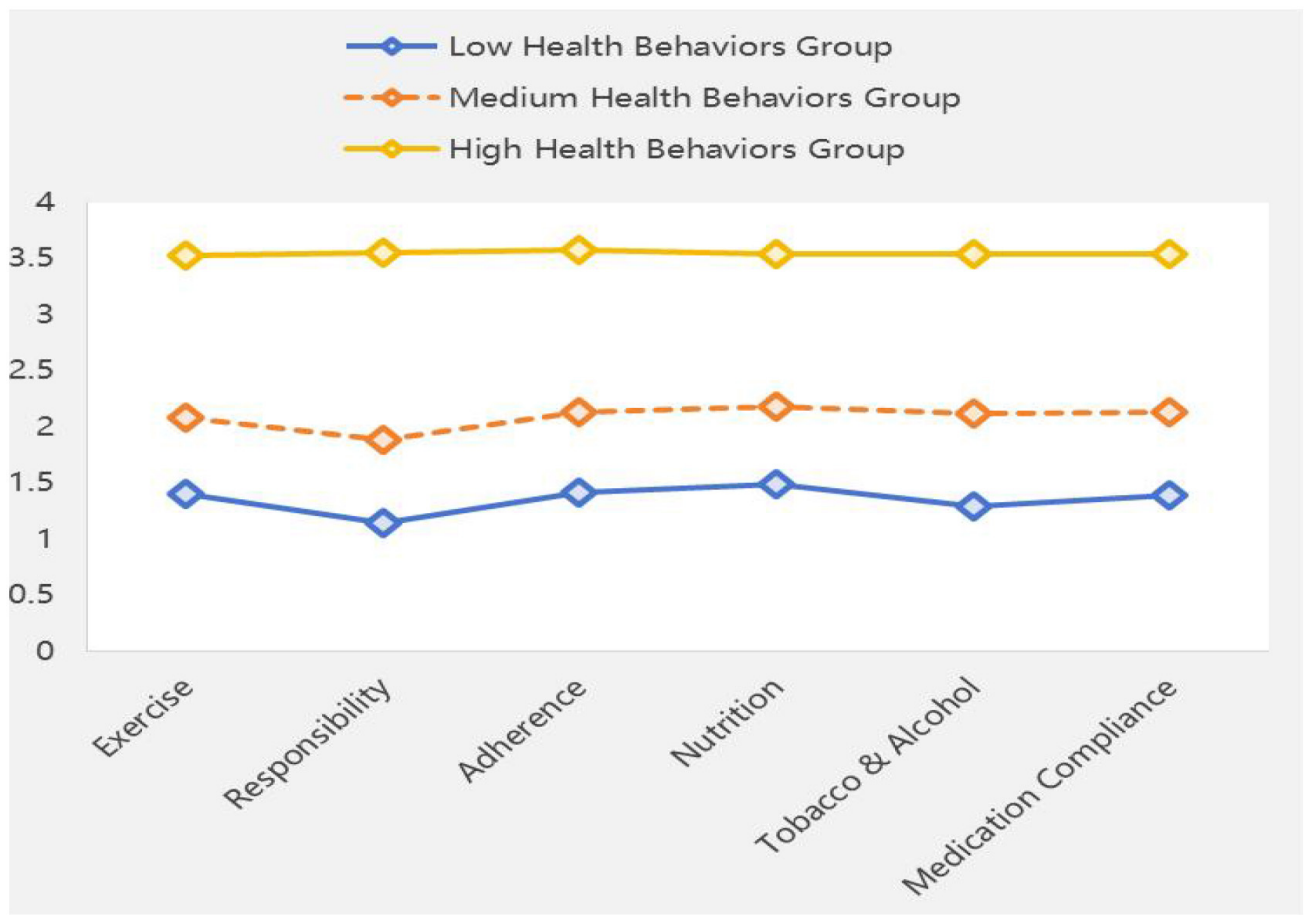
Latent profiles of health behaviors in rural patients with ischemic stroke.

On this basis, the potential profiles of the 3 potential profiles in the 6 exogenous indicators were obtained, see Figure 1, and each latent profile was named as “Low Health Behaviors Group”, “Medium Health Behaviors Group” and “High Health Behaviors Group” according to the scores of each potential profile in each dimension. As illustrated in Figure 1, the ‘Low Health Behaviors Group' scored lowest across all six health behavior dimensions (exercise, responsibility, adherence, nutrition, tobacco & alcohol, and medication compliance). Quantitatively, the estimated mean scores for the three profiles (from Mplus output) were: the Low Health Behaviors Group below 1.5 on all six dimensions (range 1.14–1.48), the Medium Health Behaviors Group between 1.89 and 2.19, and the High Health Behaviors Group between 3.53 and 3.58 (on a 0–4 scale).

### 3.4 Multifactorial logistic regression analysis of latent profiles

To identify the independent correlates of profile membership, a multinomial logistic regression was performed with the latent profile membership as the dependent variable (using the “High Health Behaviors Group” as the reference category). The results, presented in Table 5, revealed that gender, occupation, monthly household income, self-efficacy, and health knowledge were significant influencing factors of the latent health behavior profiles (*P* < 0.05). The model showed good fit (likelihood ratio χ^2^ = 307.656, *df* = 18, *p* < 0.001; Nagelkerke R^2^ = 0.643). All variance inflation factors (VIFs) were below 1.5, indicating no significant multicollinearity.

**Table 5:** Logistic regressions for differently health behaviors profiles (*N* = 378).

Variables	Category 1 (ref. category 3)			Category 2 (ref. category 3)		
	*OR*	95%*CI*	*P*	*OR*	95%*CI*	*P*
Gender	
Male	36.63	9.81–136.83	< 0.001	9.41	2.69–32.85	< 0.001
Female (Ref.)						
Occupation	
Unemployed	22.30	2.41–206.73	0.006	12.14	1.39–105.77	0.024
Physical labor	1.09	0.19–6.11	0.925	1.48	0.29–7.63	0.642
Mental labor	0.49	0.08–2.94	0.437	0.77	0.15–4.10	0.763
Others (ref.)						
Monthly income (RMB)	
< 1,000	1.46	0.21–10.14	0.702	2.15	0.34–13.50	0.414
1,001~3,000	4.74	0.53–42.20	0.163	32.50	4.14–255.12	0.001
3,001~6,000	10.76	1.16–100.16	0.037	18.01	2.16–150.10	0.008
>6,000 (ref.)						
**Self-efficacy**	0.29	0.17–0.50	< 0.001	0.34	0.20–0.58	< 0.001
**Health knowledge**	0.51	0.38–0.68	< 0.001	0.52	0.39–0.69	< 0.001

Category 1: Low Health Behaviors Group; Category 2: Medium Health Behaviors Group; Category 3: High Health Behaviors Group. Ref indicates the reference category. Reference categories: High Health Behaviors Group for outcome; female for gender; ‘Others' for occupation; >6,000 RMB for monthly income.

Specifically, compared to females, male gender was significantly associated with both unfavorable profiles: the “Low Health Behaviors Group” (OR=36.63, 95% CI: 9.81–136.83, *P* < 0.001) and the “Medium Health Behaviors Group” (OR=9.41, 95% CI: 2.69–32.85, *P* < 0.001). Regarding occupation, unemployed patients had significantly higher odds of belonging to the “Low Health Behaviors Group” (OR=22.30, 95% CI: 2.41–206.73, *P* = 0.006) and the “Medium Health Beh aviors Group” (OR=12.14, 95% CI: 1.39–105.77, *P* = 0.024) compared to those in the “Others” occupation category.

Compared to the high-income group (>6,000 RMB), the middle-income group (3,001~6,000 RMB) had a significantly higher odds of belonging to both the “Low Health Behaviors Group” (OR = 10.76, 95% CI: 1.16–100.16, *P* = 0.037) and the “Medium Health Behaviors Group” (OR = 18.01, 95% CI: 2.16–150.10, *P* = 0.008). Notably, the low-income group (1,001~3,000 RMB) exhibited a particularly strong association with membership in the “Medium Health Behaviors Group” (OR = 32.50, 95% CI: 4.14–255.12, *P* = 0.001), which was the strongest association observed among all income comparisons.

Furthermore, self-efficacy and health knowledge emerged as influence factors. For each one-unit increase in self-efficacy, the odds of belonging to the “Low” and “Medium” health behavior groups decreased by 71% (OR = 0.29, 95% CI: 0.17–0.50, *P* < 0.001) and 66% (OR = 0.34, 95% CI: 0.20–0.58, *P* < 0.001), respectively. Similarly, each one-unit increase in health knowledge was associated with an approximately 48% reduction in the odds of belonging to these two groups (“Low” OR = 0.51, 95% CI: 0.38–0.68; “Medium” OR = 0.52, 95%CI: 0.39–0.69, all *P* < 0.001).

**Figure 2:**
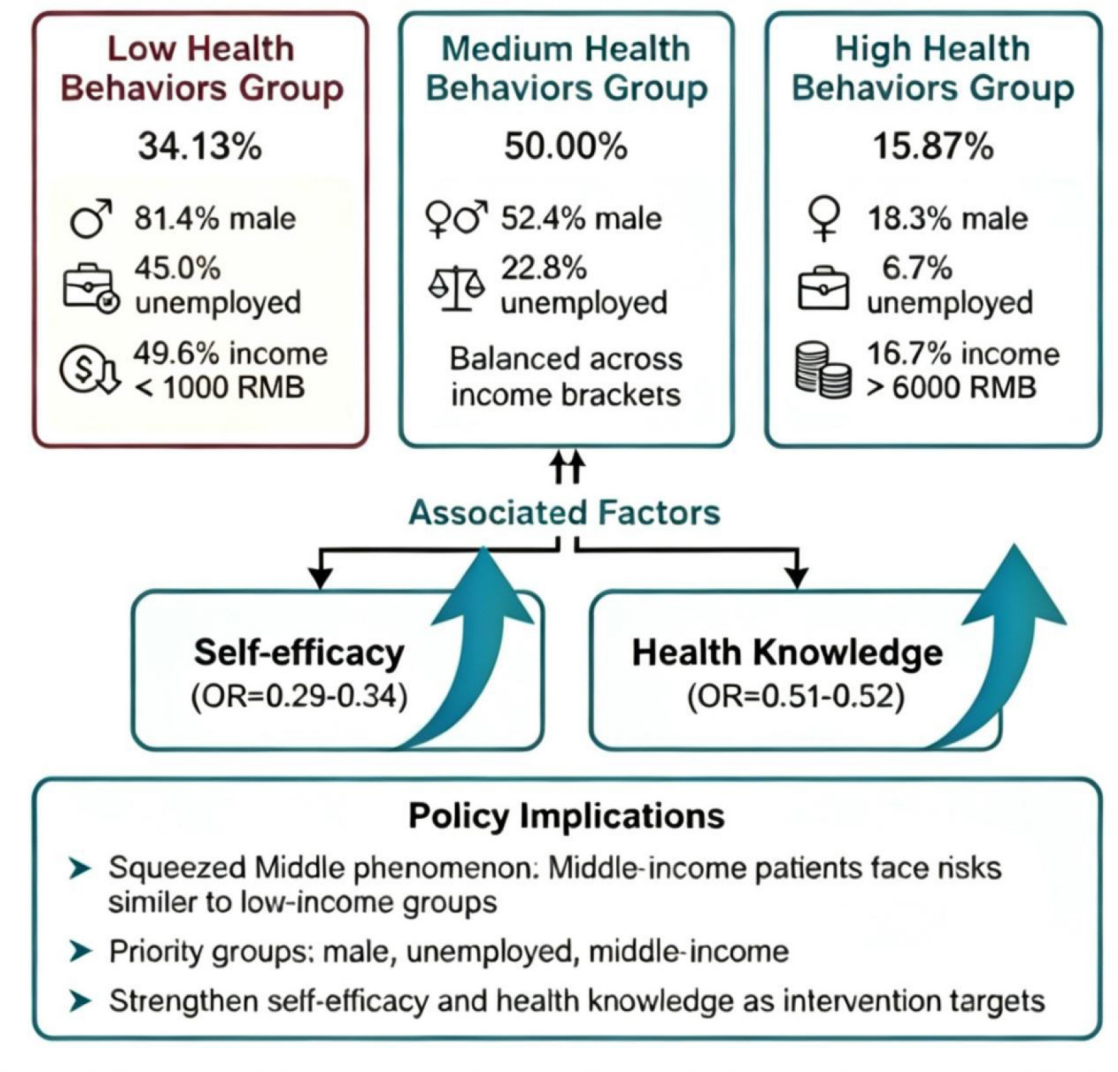
Conceptual summary of health behavior profiles and policy implications among rural patients with ischemic stroke.

To visually summarize the three identified health behavior profiles, their key sociodemographic characteristics, and the factors associated with favorable profile membership, a conceptual diagram was constructed (Figure 2).

## 4 Discussion

To our knowledge, rarely have studies applied latent profile analysis to model the multivariate health behavior patterns specifically among rural Chinese ischemic stroke survivors. Therefore, this study provides preliminary empirical evidence of distinct health behavior profiles in this population. The key finding is the identification of three distinct behavioral profiles: the “Low Health Behaviors Group” (34.13%), the “Medium Health Behaviors Group” (50.00%), and the “High Health Behaviors Group” (15.87%). This classification, supported by excellent model fit indices, underscores the significant heterogeneity within this population. Given that over 84% of patients classified into medium or low behavior profiles, our findings underscore the urgent need for improved secondary prevention strategies in rural China. From a clinical stroke perspective, these findings highlight that a substantial majority of rural survivors are not meeting evidence-based behavioral recommendations for secondary prevention. This pattern has been associated with elevated risk of recurrent stroke and poorer long-term outcomes in previous longitudinal studies (6, 9).

Male gender emerged as a strong correlate: it was both statistically significant and strongly associated with the Low Health Behaviors Group (OR = 36.63) and also with the Medium Health Behaviors Group (OR = 9.41), although the effect size was considerably larger for the Low group. This aligns with research on masculinity and health behaviors. Traditional masculine norms often discourage health-seeking behaviors (37). In rural Chinese contexts, where men frequently serve as primary breadwinners, stroke-related disability may create particular psychological conflicts that further inhibit health behaviors adoption (38). Qualitative studies have documented how inability to fulfill the breadwinner role challenges core aspects of masculinity (39). Thus, viewing all male patients as a homogeneous high-risk group may mask behavioral heterogeneity. Gender-sensitive interventions should precisely target patients with comprehensively poorer patterns (particularly the Low group), rather than all men. However, because of the cross-sectional design, these associations do not imply causality and should be interpreted as correlational.

The socioeconomic gradient revealed a non-linear pattern. Unemployment consistently predicted unfavorable profiles, likely through economic hardship and psychological distress. The income-behavior association was non-linear: middle-income (3,001–6,000 RMB) predicted both Low and Medium groups, while lower-middle income (1,001–3,000 RMB) showed the strongest association with the Medium group (OR = 32.50). This supports the “squeezed middle” concept in health disparities research (40), with similar observations in other settings (41). These patients often fall outside social safety nets while facing substantial out-of-pocket healthcare expenses, creating financial pressures that compromise their capacity for self-management (42). This group, encompassing both lower-middle and middle-income individuals, may be unable to fully benefit from poverty alleviation policies targeting extreme poverty, yet struggle to afford out-of-pocket expenses for long-term disease management, trapping them in a dilemma regarding investments in health behaviors. This indicates that economic support policies must go beyond simple poverty line classifications and focus on addressing the specific vulnerabilities of this broad “missing middle” group, particularly those in the lower-middle income range who exhibited the strongest association. Interventions aimed at reducing stroke-related health inequities must consider not only the poorest segments of the rural population but also those with moderate economic constraints who face unique barriers to adopting healthy behaviors. However, the wide confidence intervals for some income-related estimates (e.g., OR = 32.50, 95% CI: 4.14–255.12) suggest model instability; therefore, these specific findings should be interpreted with caution and require replication in larger samples.

The strong associations of self-efficacy and health knowledge with favorable health behavior profiles offer suggestive insights for intervention development. Self-efficacy was strongly associated with profile membership: each unit increase corresponded to 66%−71% lower odds of belonging to unfavorable behavioral profiles. This finding is consistent with social cognitive theory's emphasis on self-efficacy as a fundamental determinant of health behaviors adoption and maintenance (23). Similarly, health knowledge was substantially associated with favorable health behavior profiles, highlighting the potential value of literacy-appropriate health education in resource-limited settings (43). These modifiable factors represent promising targets for interventions across all identified profiles, suggesting that community-based programs combining skill-building with knowledge enhancement could effectively promote improved self-management.

The clinical implications of our findings support a stratified approach to secondary prevention. For the “Low Health Behaviors Group”, characterized by male gender, unemployment, and limited psychosocial resources, resource-intensive interventions addressing both behavioral and socioeconomic barriers could be considered. This might include community health worker support, medication subsidies, and integrated mental health services (44). Organizational interventions, such as case management, have been shown to improve blood pressure control in secondary stroke prevention (45). For the larger “Medium Health Behaviors Group”, scalable, brief interventions focusing on action planning, problem-solving, and habit formation may efficiently promote behaviors maintenance and improvement (46).

To translate these stratified recommendations into practice within the rural Chinese context, careful attention to feasibility is paramount. For the “Low Health Behaviors Group”, even resource-intensive approaches such as one-on-one support by community health workers would need to be carefully designed for sustainability, potentially through targeted task-sharing within the existing primary care system. To enhance self-efficacy and health knowledge, scenario-based skill training, peer-led education groups, and structured telehealth follow-up could be considered; mobile health (mHealth) platforms could be explored to address geographic barriers (47). Peniche et al. found that telehealth interventions demonstrate utility in stroke secondary prevention, particularly in blood pressure reduction (45). For the “Medium Health Behaviors Group”, scalable low-cost strategies such as SMS-based reminders, visual educational materials, and group-based counseling during follow-up visits could be considered. To ensure feasibility in rural China, task-sharing models might be considered for integration into existing primary care systems, pending feasibility studies. Low-cost mHealth tools (e.g., SMS reminders, educational videos) might help extend reach without requiring substantial additional resources (48). For middle-income patients, financial mechanisms such as sliding-scale fees could be considered to improve affordability. Ultimately, any program would need to be developed in partnership with local communities and healthcare providers to ensure cultural relevance and alignment with existing workflows and resource capacities. Future research should examine the temporal stability of these profiles and develop tailored interventions that address profile-specific barriers and leverage profile-specific strengths. By identifying distinct behavioral subgroups within an underserved rural population, this study offers preliminary evidence for developing targeted interventions that can effectively reduce stroke-related health disparities.

## 5 Clinical implications

These findings suggest that a one-size-fits-all approach to secondary prevention may be insufficient. Patients in the Low Health Behaviors Group (predominantly male and unemployed) might benefit from more intensive support, while those in the Medium group could be managed with scalable strategies. Stroke education programs might benefit from incorporating skill-building components to enhance self-efficacy and health knowledge. Consideration of integrating such behavioral strategies into routine stroke care may help improve outcomes in rural populations with limited access to specialized services.

## 6 Conclusion

This study identified three distinct latent profiles of health behaviors among rural patients with ischemic stroke in China, revealing significant heterogeneity that underscores the limitation of one-size-fits-all interventions. The findings of this study suggest which subgroups and modifiable factors might be prioritized in future intervention studies. Tailored interventions targeting low and medium health behavior groups should be prioritized. Key associated factors included sociodemographic characteristics and, more importantly, modifiable psychological assets (self-efficacy, health knowledge). These findings offer preliminary evidence that may serve as a reference for future intervention studies targeting behavioral and psychosocial determinants, with the ultimate goal of reducing stroke recurrence and improving outcomes in rural China.

## 7 Strengths and limitations

To our knowledge, rarely have studies applied latent profile analysis to health behaviors among rural patients with ischemic stroke in China. The findings provide novel evidence for the substantial heterogeneity in behavioral patterns within this vulnerable population, thereby laying a crucial foundation for developing precise and tailored secondary prevention strategies. However, several limitations warrant consideration. First, self-reported data may introduce recall bias. Moreover, because many participants required assisted questionnaire completion (despite standardized verbatim administration), social desirability bias and potential interviewer influence cannot be fully ruled out. Readers should consider these limitations when interpreting the health behavior data. Second, the use of convenience sampling in outpatient settings may have introduced selection bias. Patients who regularly attend follow-up visits might have better health behaviors than those who do not, potentially overestimating the overall behavioral levels in the broader rural stroke population. Additionally, our sample was drawn from only one province (Liaoning), which limits generalizability to rural areas in central and western China. Third, the cross-sectional nature of this study precludes the establishment of causal relationships and limits deeper mechanistic exploration. Specifically, while we identified key structural barriers and psychosocial factors as independent correlates, our analysis did not test for potential interactions or moderating effects between them (e.g., whether resource constraints attenuate the benefit of high self-efficacy). Fourth, the study did not include several clinical variables that may influence health behaviors, such as stroke severity (e.g., NIHSS score), time since stroke onset, or the presence of specific post-stroke complications (e.g., depression or cognitive impairment). The absence of these potential confounders limits our ability to fully disentangle the determinants of the observed behavioral profiles. Fifth, some multinomial logistic regression estimates (e.g., unemployment in the Low Health Behaviors Group) had wide confidence intervals, likely due to small subgroup sizes in the reference categories (e.g., the ‘Others' occupation group had only 55 participants). These estimates should be interpreted with caution, and replication in larger samples is warranted. Future longitudinal studies with larger, more representative samples are needed to validate the stability of the identified profiles, to investigate their predictive relationship with critical outcomes such as stroke recurrence and mortality, and to unravel the complex interdependencies among determinants.

## Data Availability

The raw data supporting the conclusions of this article will be made available by the authors, without undue reservation.
